# Liver Matrix Stiffening Modulates Tumor-Associated Hepatocyte Polyploid Homeostasis via Piezo1/RUNX2/Anillin Mechanosensitive Axis

**DOI:** 10.3390/ijms27114685

**Published:** 2026-05-22

**Authors:** Xinyi Luo, Yifan Zhang, Yiquan Lu, Nan Wang, Fengjie Hao, Yongjun Chen, Xiaochun Fei, Junqing Wang

**Affiliations:** 1 Department of General Surgery, Ruijin Hospital, Shanghai Jiao Tong University School of Medicine, 197, Rui Jin Er Road, Shanghai 200025, China; 2 Department of Internal Medicine III, University Hospital RWTH Aachen, 52074 Aachen, Germany; 3 Department of Pathology, Ruijin Hospital, Shanghai Jiao Tong University School of Medicine, 197, Rui Jin Er Road, Shanghai 200025, China

**Keywords:** liver matrix stiffness, mechanosensitive axis, polyploid hepatocyte homeostasis, hepatocellular carcinoma, nuclear translocation

## Abstract

The human liver is a polyploid organ, dominantly featured by a high proportion of binuclear polyploid hepatocytes. Our recent study demonstrates that decline of the abundance of binuclear hepatocytes (ABH) plays a critical role in contributing to Hepatocellular carcinoma (HCC) formation, involving the cytokinesis regulator Anillin. However, the relevance between liver stiffness and the acquired ABH attenuation remains unclear. In this study, we set a mechanical environment gel with different gradients to simulate different liver stiffness environments, combined with the paired paracancerous liver tissues from real-world patients with HCC who underwent radical surgery. A mechanosensitive Piezo1/RUNX2/Anillin axis was discovered. As observed, the decline of ABH in paracancerous liver tissues is a noteworthy measurable value for tumor formation, correlated with the extent of liver matrix stiffness and dismal phenotypes. A stiffened culture environment may promote quick polyploid attenuation of hepatocytes, accompanied by high expression of Piezo1, a critical mechanosensitive ion channel, and a consequential nuclear translocation of RUNX2. Importantly, RUNX2 functions as an upstream transcription factor of Anillin. Regulating Piezo1/RUNX2 or using Piezo1 agonist remarkably affected Anillin expression and hepatocyte polyploidy homeostasis. Thus, we propose that the Piezo1/RUNX2/Anillin axis transduces the microenvironment mechanical signal from liver stiffening and impairs hepatocyte polyploidy homeostasis in HCC formation.

## 1. Introduction

Hepatocytes play predominant roles in the liver by exerting complex functions, and their malignant transformation commonly results in the incidence of hepatocellular carcinoma (HCC) [1,2]. As acknowledged, matrix stiffness is a pivotal biological feature and driving factor of human malignancies, in breast, pancreas, and liver cancer [3]. Uncontrolled intrahepatic pathological impairment may induce gradually worsening liver fibrosis and cirrhosis, which lead to composite microenvironment changes, including matrix stiffness and a variety of relevant mechanical stress and pressure intrahepatic [4]. However, the exact mechanisms of liver matrix stiffness inducing hepatocyte transformation and liver tumorigenesis have not been fully illustrated.

On the other hand, the human somatic chromosomes are basic diploid (2n), while a few human organs, like the myocardium, bone marrow, and liver, may physiologically generate polyploid cell populations (4n or 8n) to meet the necessities of cellular functions or the particular physiological conditions [5]. Accumulating evidence has revealed that the liver is a cellular polyploidization organ, with an abundance of up to 50% and 90% of adult and mouse polyploid hepatocytes, respectively [6,7]. Hepatocyte polyploidization is a dynamic change that originates in postnatal mammals from the diploid hepatocytes during the process of mitosis, mainly through the method of ‘cytoplasmic division failure’, and is predominantly composed of binuclear hepatocytes (4n) [8]. The homeostasis of the binuclear hepatocyte serves the liver to adapt to physical development, DNA damage repair, and liver function maintenance, and provides an important protective mechanism for the liver to resist the occurrence of HCC [9]. It has been reported that the pathological factors, like intrahepatic chronic inflammation and rapid injury induced by carbon tetrachloride ordiethylnitrosamine (DEN), may impair the homeostasis of hepatocytes and cause polyploid attenuation, as an important cellular event before the occurrence of HCC, leading to a sharp decrease in the abundance of polyploid liver cells [10].

In our previous study, we proposed and introduced the concept of abundance of binuclear hepatocytes (ABH) to describe the overall distribution of polyploid hepatocytes in the paracancerous liver region [11]. By retrospectively studying the short-term recurrence of the HCC patients who had received radical operation, we found that ABH attenuation in the paracancerous liver tissues is an independent risk factor for clinical recurrence of HCC, which suggests the change in the binuclear hepatocyte status of the intrahepatic environment is associated with tumor formation [12]. Meanwhile, we also validated a series of effectors particularly affecting ABH through modulating cytokinesis failure, including the Actin Binding Protein Anillin, the transcription factor E2F7, and noncoding RNAs like Anril [13,14,15]. Even though some of the mechanisms in polyploid attenuation of hepatocyte have been revealed, the regulatory relationship between the intrahepatic microenvironment, especially the liver matrix stiffness, and the alteration of ABH during HCC formation has not been fully illustrated.

To resolve the above yet uncertain mechanism, we focused on the mechanotransduction in the matrix [16], which we believe may exert the function in recognizing the mechanical stimulation and transducing the extracellular mechanical stimulation into the hepatocytes as biochemical signals. We simulated the liver matrix by generating a stiff substrate in vitro, and then explored the impairment of mechanical force from the matrix on the polyploidy status of hepatocytes. During this process, Piezo-type mechanosensitive ion channel component 1 (Piezo1) was recognized as the critical membrane sensor, which presented a high expression in hepatocytes. Previously, most studies of Piezo1 were commonly concentrated on the stromal cells, particularly hepatic stellate cells (HSCs). Here, for the first time, we innovatively discussed this mechanosensitive molecule in the process of hepatocyte polyploid attenuation. We discovered that Piezo1 activation may significantly drive the nuclear translocation of the transcription factor RUNX2, and intriguingly, lead to a consequential increase in Anillin. Along with the functional change in the potential Piezo1/RUNX2/Anillin axis, the abundance of the binuclear polyploid hepatocytes was impacted. Based on our in vitro fundings, we are confident that the liver matrix stiffening stimulates the mechanosensitive Piezo1/RUNX2/Anillin axis in the microenvironment of the liver, and further modulates the polyploidy homeostasis of hepatocytes during tumorigenesis. This mechanism for HCC formation deserves intensive investigation for an in-depth understanding of the HCC tumorigenesis involved with the particular stiffening in the liver.

## 2. Results

### 2.1. Liver Matrix Stiffening May Contribute to ABH Attenuation in the Paracancerous Liver Tissues Associated with HCC Susceptibility

The measurement to grade the liver matrix stiffness was carried out, and 78 of 142 cases (35 of whom relapsed shortly after R0 radical resection surgery) were classified as ‘lower stiffness’, and the other 64 were classified as ‘higher stiffness’.

**Figure 1:**
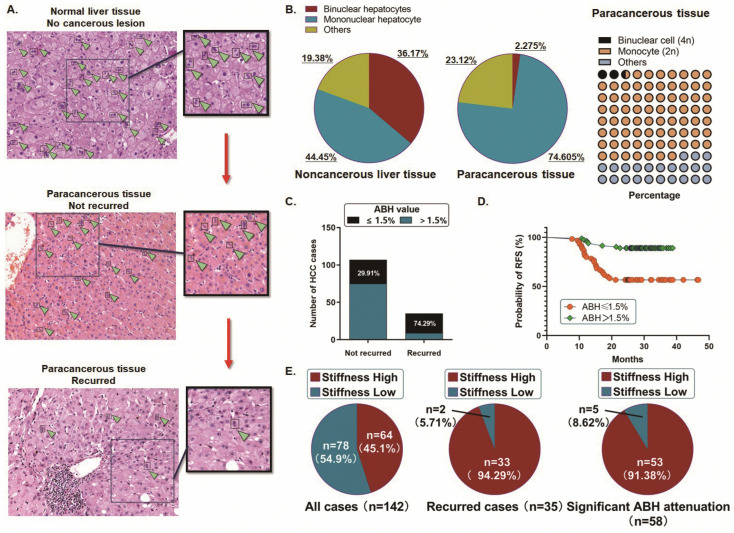
The correlation between liver stiffness, abundance of binuclear polyploid hepatocytes (ABH), and susceptibility (recurrence) of HCC. (**A**) The number of binucleated 4n liver cells (indicated by the arrow) in the adjacent tissues of HCC patients is significantly reduced compared to normal liver tissues, and the most significant reduction in the number of binucleated 4n liver cells is observed in the adjacent tissues of susceptible HCC (recurrence) patients. (**B**) A description of the overall state of polyploid liver cells in adjacent liver tissue using dual nucleus 4n liver cells. The ABH ranges from 1.1% to 5.25% (mean: 2.28%; median: 2.23%; SD: 1.370). (**C**) Patients with significantly reduced ABH are more susceptible to HCC, with 74.29% of recurrent cases showing a significant decrease in ABH, compared to only 29.91% of non-recurrent cases (*p* < 0.01). (**D**) The Kaplan–Meier plot showed a significant decrease in RFS rate in patients with significant ABH attenuation (*p* < 0.01). (**E**) 45.1% (64/142) of the 142 cases showed significant hardening of the adjacent tissues; 94.29% (33/35) of recurrent cases were from the ‘high liver hardness’ group; 91.38% (53/58) of ABH attenuation was associated with high liver hardness (*p* < 0.01).

According to the pathological examination, combined with the improved ImageJ software, version 1.54 algorithm reported [12], all the paracancerous liver tissues and 43 samples from the normal liver tissues were investigated. The ABH was proposed to describe the overall state of polyploid hepatocytes in the paracancerous microenvironment. As the HE staining detection demonstrated, the number of binuclear polyploid hepatocytes generally decreased from normal liver to adjacent tissue to HCC, with the most significant decline observed in patients with short-term postoperative recurrence (Figure 1A), and the ABH ranged from 1.1% to 5.25% (mean: 2.28%; median: 2.23%; SD: 1.370) (Figure 1B). In total, 74.29% (26/35) of the recurrence cases showed a significant decrease in ABH (by using the cutoff value: ≤ 1.5%) (Figure 1C); ABH attenuation was associated with shorter recurrence-free survival (RFS) (*p* < 0.01) (Figure 1D); 94.29% (33/35) of recurrent cases were from the ‘higher stiffness’ group; 91.38% (53/58) of cases with ABH attenuation were associated with high liver matrix stiffness (Figure 1E). The above prompt suggests that the inherent mechanical force generated by liver matrix stiffness is related to the attenuation of polyploid hepatocytes, which is an important clinical value of HCC susceptibility.

### 2.2. Piezo1 Expression Is Associated with the Extent of Liver Matrix Stiffness in the Paracancerous Liver Tissues, Along with the ABH Attenuation

**Figure 2:**
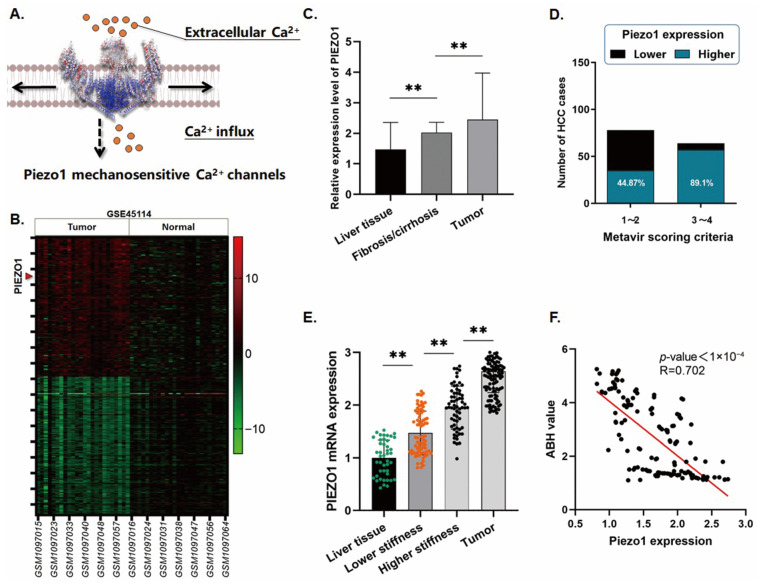
Association between Piezo1 and the extent of liver matrix stiffness and ABH in the paracancerous liver tissues. (**A**) The diagram of the working mechanism of Piezo1 located on the hepatocyte membrane in mediating the second messenger Ca^2+^ influx. (**B**) The heatmap generated from the GSE45114 dataset, containing 49 liver cancer-related samples sequencing data, shows a significant upregulation of Piezo1 in HCC. (**C**) Analysis of the NCBI-GEO database datasets (GSE14520, GSE14323, and GSE6764). Piezo1 gradually increased in the pathological progression of ‘liver tissue’ to ‘liver fibrosis/sclerosis’ and to ‘tumor’ (** *p* < 0.01). (**D**) Comparing with 43 normal liver tissues from our medical center, 44.87% (35/78) of cases from the ‘lower stiffness’ group, and 89.1% (57/64) of cases from the ‘higher stiffness’ group were detected with a high expression of Piezo1. (**E**) Piezo1 upregulation showed much more significance in the ‘higher stiffness’ group, in comparison with the ‘lower stiffness’ group (** *p* < 0.01). (**F**) Expression of Piezo1 was significantly correlated with the attenuation of ABH, in the context of liver matrix stiffening.

Piezo1 is an ion channel distributed on the mammalian cell membrane responsible for regulating cellular Ca^2+^ influx, as the illustration presents (Figure 2A). As shown in the heatmap, which was generated by analyzing the sequencing data of 49 liver cancer-related samples from the GSE45114 dataset, the expression of Piezo1 was significantly upregulated in HCC (Figure 2B). Furthermore, we explored the expression trend of Piezo1 by comparing three NCBI-GEO datasets (GSE14520, GSE14323, and GSE6764). As observed, the expression of Piezo1 gradually increased in the pathological progression of ‘liver tissue’ to ‘liver fibrosis/cirrhosis’ and to ‘tumor’ (Figure 2C). We propose that the upregulation of Piezo1 is consistent with the aggravated liver stiffening.

Similarly, in comparison with 43 normal liver tissues from our medical center, 44.87% (35/78) of cases from the ‘lower stiffness’ group, and 89.1% (57/64) of cases from the ‘higher stiffness’ group were detected with a high expression of Piezo1. Notably, the upregulation of Piezo1 in the ‘higher stiffness’ group presented significance in contrast to the ‘lower stiffness’ group (Figure 2D,E). Simultaneously, the change in Piezo1 expression showed a significant correlation with the attenuation of ABH, in the context of liver matrix stiffening (*p <* 0.0001 R = 0.702) (Figure 2F).

### 2.3. High Piezo1 Expression in Paracancerous Hepatocytes Is Associated with Dismal Tumor Phenotypes of HCC Patients, Especially Rapid Tumor Recurrence

The clinicopathologic features of the 142 cases were analyzed statistically to discuss their correlation with Piezo1 expression. According to the data shown in Table 1, there was no significant correlation observed between the paracancerous expression of Piezo1 and the patient’s general information, including age, gender, or even the instant virus control status. However, the upregulation of Piezo1 was remarkably related to unsatisfactory clinical phenotypes, like larger tumor size (*p* < 0.05), higher Alpha-fetoprotein (AFP) and PIVKA-II quantity (*p* < 0.05), more advanced TNM stages (*p* < 0.05), and severe microsatellite lesion formation (*p* < 0.05). It is worth noting that in the cases with higher paracancerous Piezo1 expression, a higher frequency of tumor recurrence within 2 years after surgery was observed, which strongly indicated that the instant status of Piezo1 expression paracancerously is associated with the recurrence of HCC, which proposes that Piezo1 upregulation in hepatocytes is a probable indicator of HCC tumorigenesis based on the uncontrollable changes in the intrahepatic microenvironment. This may also explain the irrelevance between Piezo1 and the intrahepatic virus control status, since the actual status of liver matrix stiffness is commonly difficult to reverse, even though the virus status could be controlled by antivirus therapy when the fibrosis or cirrhosis is firmly formed.

### 2.4. Stiffening the Culture Environment Leads to Piezo1 Upregulation, Accompanied by the Attenuation of Binuclear Polyploid Hepatocytes

**Figure 3:**
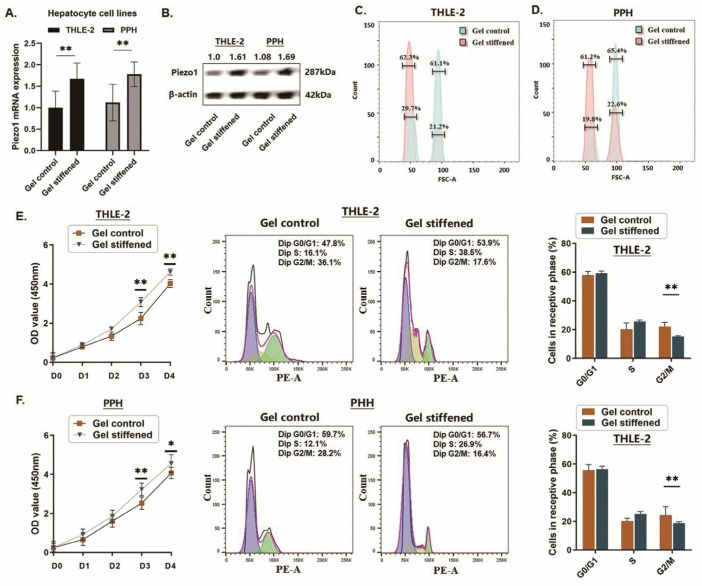
Association between Piezo1 and the extent of liver matrix stiffness and ABH in the paracancerous liver tissues. (**A**) The RT-qPCR assay indicated that the hepatocytes (THLE-2 and PHHs), which were incubated on the stiffened gel, presented a higher expression of Piezo1 with significance (** *p* < 0.01). (**B**) The Western blot analysis showed a significant upregulation of Piezo1 at the protein level in hepatocytes, which were incubated in the stiffened microenvironment. (**C**,**D**) The flow cytometry analysis was conducted. The composition of hepatocytes on the stiffened soft gel presented a higher ratio of binuclear polyploid hepatocytes and fewer diploid cells. (**E**,**F**) The CCK8 assay indicated that both THLE-2 cells and PHHs incubated in the stiffened microenvironment presented partial significance in cell proliferation activation, and the contemporary flow cytometry analysis demonstrated that an obvious decrease in the G2/M phase was observed in these treated hepatocytes (* *p* < 0.05, ** *p* < 0.01).

Both THLE-2 immortal hepatocytes and PHHs were cultured on the stiffened environment gel. By extracting the total RNA of the cultured cells, the Piezo1 expression at the mRNA level was detected. Compared with the control cells, which were incubated on the soft substrate gel, the cells incubated on the stiffened gel presented a significantly higher expression of Piezo1 (Figure 3A). A similar result was obtained by the Western blot assay which showed that Piezo1 was upregulated in hepatocytes incubated on the stiffened gel (Figure 3B). Intriguingly, as the flow cytometry analysis showed, the cell composition of the hepatocytes on the stiffened soft gel processed a higher ratio of binuclear polyploid hepatocytes and fewer diploid cells, compared with the soft gel environment (Figure 3C,D). Given the aforementioned ‘cytokinesis failure’ mechanism in hepatocyte polyploidization, we explored the cell growth ability and the cell cycle distribution of the hepatocytes according to the stiffness status for cell incubation. The cell proliferation activity was trending to increase in the cells incubated on the stiffened gel, with a partial significance in these two hepatocyte lines. Notably, an obvious decrease in the G2/M phase was observed, whereas the cell distribution changes in the G0/G1 and S phases presented no significance. These findings indicated that more hepatocytes had broken through the static of the cytokinesis and polyploid attenuation occurred in the stiffened environment (Figure 3E,F). We believe that the difference in the microenvironment stiffness may affect the polyploidy homeostasis of hepatocytes by influencing the cytokinesis process, which is consistent with the viewpoint of the published literature [8,17,18].

### 2.5. The Stiffened Microenvironment or the Specific Agonist Facilitates the Calcium Influx Mediated by the Activated Piezo1 Ion Channel

**Figure 4:**
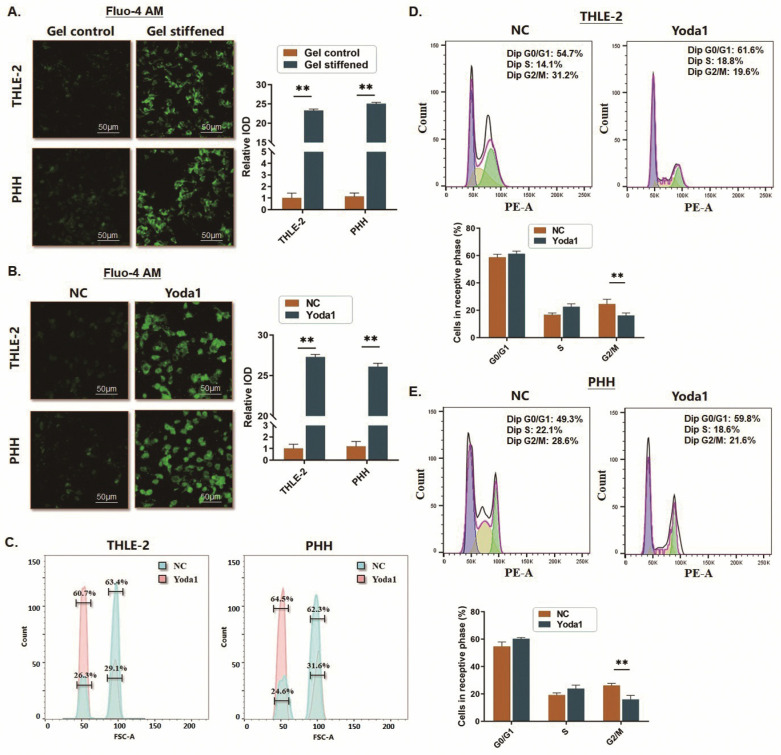
Stiffened microenvironment and Piezo1 agonist Yoda1 facilitated the activity of the Piezo1 ion channel. (**A**) The Fluo-4 AM fluorescence assay was carried out. The THLE-2 cells and PHHs presented a significant increase in Ca^2+^ influx when incubated in the stiffened microenvironment (** *p* < 0.01). (**B**) Both the THLE-2 cells and PHHs were treated with Yoda1, and the Fluo-4 AM fluorescence assay showed a significant elevation of Ca^2+^ influx by activating Piezo1 (** *p* < 0.01). (**C**) According to the flow cytometry analysis, after Yoda1 treatment, the composition of binuclear polyploid hepatocytes was increased in both THLE-2 cells and PHHs. (**D**,**E**) Representative flow cytometry histograms showing DNA-content distribution in THLE-2 or PHH cells treated with NC or Yoda1. A trend of decrease in the G2/M phase was observed in the hepatocytes treated with Yoda1, which proposed a remarkable influence of the Piezo1 activation in the polyploidy homeostasis of hepatocytes (** *p* < 0.01). The black outline indicates the total DNA-content profile, while the colored fitted areas represent different diploid cell-cycle populations: purple, diploid G0/G1 phase; yellow, diploid S phase; and green, diploid G2/M phase.

To make sure the upregulation of Piezo1 could exert the exact signal transduction, we estimated the Ca^2+^ influx status by co-culturing the hepatocytes under Ca^2+^ conditions. According to the observation based on Fluo-4 AM fluorescence detection, a significant increase in Ca^2+^ influx in the THLE-2 cells and PHHs cultured on stiffened gels was observed compared to the cells on soft gels (Figure 4A).

Similarly, when we treated the hepatocytes on the soft gel with Yoda1, an agonist of Piezo1, a significant activation of Piezo1 was observed according to the remarkable increase in Ca^2+^ influx into the hepatocytes (Figure 4B). Notably, after a 72 h Yoda1 treatment, the ratio of binuclear polyploid hepatocytes was obviously declined compared with the control group, accompanied by a trend of the cell cycle decreasing in the G2/M phase (Figure 4C–E). This means that Piezo1 activation is a pivotal factor influencing the polyploidy homeostasis of hepatocytes.

### 2.6. Piezo1 Impacts the Translocation of RUNX2

**Figure 5:**
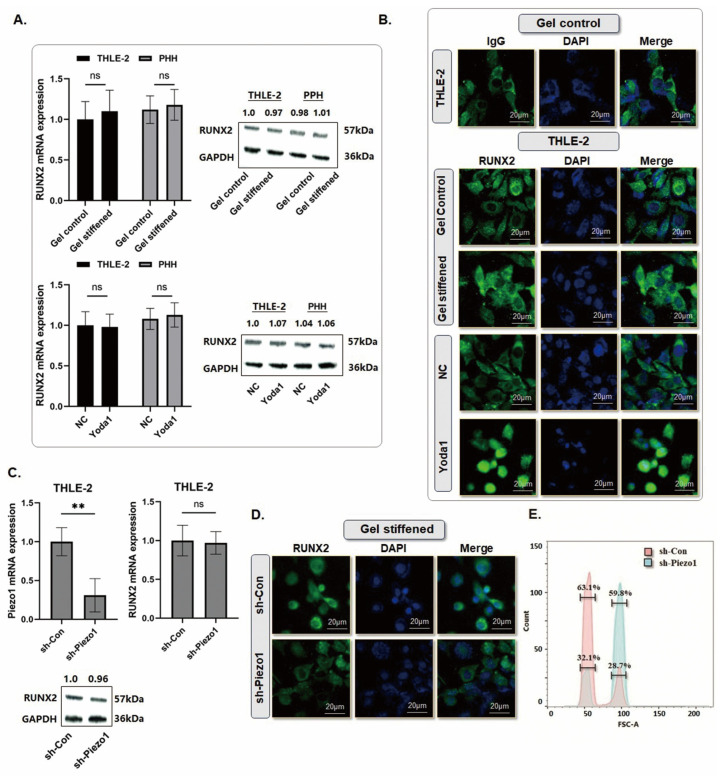
Piezo1 facilitated the nucleus translocation of RUNX2. (**A**) According to the RT-qPCR assay and Western blot analysis, the expression of RUNX2 in the hepatocytes showed no significant change, treated with neither the stiffened microenvironment nor Yoda1. (**B**) As the immunofluorescence detection demonstrated, taking the THLE-2 cells as an example, a remarkable increase in RUNX2 was observed when Piezo1 was activated. (**C**) By knocking down Piezo1 through the shRNA transfection (** *p* < 0.01), no significant expression change was detected in RUNX2 expression. (**D**) The knockdown of Piezo1 significantly suppressed the nuclear translocation of RUNX2 in the THLE-2 cells. (**E**) The knockdown of Piezo1 resulted in an obvious decrease in the binuclear polyploid hepatocytes.

Considering the influx of Ca^2+^ mediated by Piezo1, we focused on the large body of literature concerning Ca^2+^-related signaling [19,20,21], and noticed that RUNX2 might be a downstream effector of Piezo1, even though the related reports on hepatocytes or HCC were limited. As we observed, the expression of RUNX2 in the hepatocytes showed no significant change, neither on the stiffened gels nor when treated by Yoda1 (Figure 5A). However, taking the THLE-2 cells as the object, a remarkable translocation of RUNX2 into the nucleus was observed by the immunofluorescence detection (Figure 5B). To investigate if RUNX2 translocation was influenced by the activation of Piezo1, the lentiviral vectors pLKO.1 containing shRNA for Piezo1 were introduced into the THLE-2 cells via lentiviral transduction, which caused a measurable decline in Piezo1. As observed, knocking down Piezo1 resulted in no significant expression change in RUNX2 (Figure 5C). However, when cultured on the stiffened gel, the nuclear distribution of RUNX2 was obviously interfered with, and the proportion of binuclear polyploid hepatocytes declined (Figure 5D,E).

### 2.7. Piezo1activation Induces Anillin Transcription by Promoting RUNX2 Nuclear Translocation

**Figure 6:**
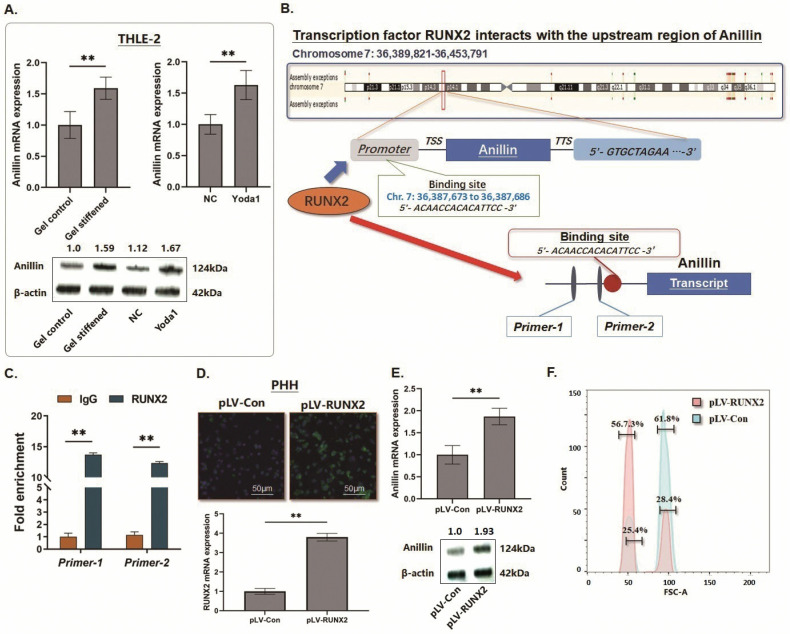
Piezo1 facilitated the nuclear translocation of RUNX2. (**A**) The expression of Anillin in the THLE-2 cells treated with a stiffened microenvironment or Yoda1 was detected by the RT-qPCR and Western blot assay. Anillin was significantly upregulated both at the mRNA and protein levels after the treatment (** *p* < 0.01). (**B**) RUNX2 was predicted as one of the potential transcription factors binding to the promoter region of the Anillin gene (5′- TGTGTGGTTGTTGGGGGTGG-3′, Chr. 7: 36389611 to 36389620). Two special primers (*Primer-1* and *Primer-2*) were designed. (**C**) The ChIP assay was conducted to investigate the interaction between RUNX2 and the promoter region of the Anillin gene. IgG was used as the negative control (** *p* < 0.01). (**D**) RUNX2 was upregulated in the PHHs by using the lentiviral vector pLV transfection (** *p* < 0.01). (**E**) Anillin expression presented a significant increase in PHHs after RUNX2 upregulation (** *p* < 0.01). (**F**) The proportion of binuclear polyploidy PHH was observed to decrease remarkably by upregulating RUNX2.

Since Anillin is described as the terminal effector of cytokinesis, we detected the expression of Anillin in the THLE-2 cells treated with different culturing stiffness or Yoda1. According to the RT-qPCR and Western blot assays, the expression of Anillin was significantly increased both at the mRNA and protein levels (Figure 6A). Based on the findings that Piezo1 influences the translocation of transcription factor RUNX2, we proposed that RUNX2 might play a critical role in promoting Anillin expression. In this study, a 3000-bp length fragment from the promoter region of the Anillin gene was captured, and a specific nucleotide sequence (5′- ACAACCACACATTCC -3′, Chr. 7: 36387673 to 36387686) potentially binding to the transcription factor RUNX2 intra-nucleus was screened with a credible significance (*p* = 3.61 × 10^−5^), by using Database of Human Transcription Factor Targets (http://bioinfo.life.hust.edu.cn/hTFtarget#!/ (accessed on 18 December 2025)) and Gene-Cloud of Biotechnology Information (GCBI, https://www.gcbi.com.cn (accessed on 20 December 2025)) (Figure 6B). In accordance with this, we conducted the ChIP assay following the instruction, and a direct interaction between RUNX2 and this short sequence upstream Anillin gene was verified (Figure 6C). As shown in Figure 6D, RUNX2 upregulation was carried out by lentiviral vector pLV transfection in PHHs, and a significant increase in Anillin expression at the mRNA level was detected (Figure 6E). Sequentially, the proportion of binuclear polyploid cells in the PHH population further decreased following the upregulation of RUNX2 (Figure 6F), suggesting that the transcriptional activity effect of RUNX2 on Anillin impairs the homeostasis of polyploid hepatocytes.

## 3. Discussion

HCC is one of the major health challenges worldwide, presenting aggressive invasiveness and high incidence of metastasis and rapid recurrence [22]. Even after receiving radical resections, short-term relapse in situ may occur in 20% of patients during the first 24 months after surgery [23,24]. Distinctive histological differentiation characteristics, specific tumor immune status, and particular tumor microenvironment landscapes form the complex background for quick tumor recurrence [25,26]; while, on the other hand, we believe that all these above factors may potentially provide important clues for understanding the mechanisms underlying HCC tumorigenesis and help to predict and prevent tumor formation.

Previous research on HCC has commonly been dedicated to discovering the explanation from the tumor tissue itself or focused on the tumor cell biology and phenotypes. Only a little attention was paid to the hepatic microenvironment and the related somatic cell phenotype changes, which may probably occur pre-tumorigenesis. Based on this point, we look inside the cellular morphological changes in hepatocytes to explore the relative signs for tumor formation.

The liver is recognized as the cellular polyploidization organ for adapting to the physiological necessities and to protect against chromosome aberration and liver tumorigenesis [27]. Quantification of the polyploid hepatocytes is prompted as a possible way for assessing HCC prognosis [7]. Referring to our published literature, the impairment of polyploid homeostasis is an important cellular pathological phenotype associated with intrahepatic microenvironment changes, and the attenuation of polyploid hepatocytes serves to engage tumorigenesis [11]. The binuclear hepatocytes composite the maximum proportion of the polyploid hepatocytes, and changes in this cell population induce the greatest impact on the homeostasis of polyploid cells.

According to the measurement of the ABH, we noticed that the instant existing paracancerous microenvironment has a significant correlation with both polyploidy status and short-term tumor recurrence. Moreover, Anillin, which plays a key role in intracellular polyploid attenuation, was validated to be increased not only in the tumor cells, but also in the paracancerous microenvironment [14]. Thus, we are confident that the illustration of the mechanism impacting polyploid homeostasis in the tumor-related microenvironment may help to understand the tumorigenesis of HCC.

Referring to our latest findings, measuring the ABH in the paracancerous liver tissue to quantify the reduction in binuclear hepatocytes could effectively evaluate the instant impairment of the polyploidy homeostasis, and a sharp decline value is an independent risk factor for short-term recurrence in HCC patients [12]. On this basis, we further verified the relationship between the extent of the liver matrix stiffness and the attenuation of polyploid hepatocytes. By expanding the samples from 142 real-world cases, we highlight that the more severe the liver matrix stiffness is, the more significant attenuation of polyploid hepatocytes is engaged, and the more frequent tumor recurrence incidence after surgery. Since liver cirrhosis and uncontrollable hepatitis are the predominant causes that lead to HCC tumor formation [28,29], we supposed that the extracellular signals from these pathophysiological changes with inherent intrahepatic mechanical forces might play important roles in modulating polyploidy homeostasis of hepatocytes.

On this view, we interrogated a series of mechanical sensors or receptors, including the Piezo family, ELKN, and TTN3, seeking a breakthrough. Notably, by exploring the GEO datasets, Piezo1 was found not only highly expressed in HCC tissues, but also presented a gradual upregulation from the normal liver tissues to the fibrosis/cirrhosis liver tissues, which indicated Piezo1 upregulation as a precancerous event generated in the liver microenvironment.

Piezo1 is a member of the Piezo mechanosensitive ion channels, which control the influx of Ca^2+^ into the somatic cells by sensing the change in extracellular mechanical force [30]. As the literature reports, this molecule is characterized as a non-selective ion channel widely distributed in mammalian cell membranes with a homotrimeric, propeller-shaped transmembrane structure, by which Piezo1 senses the deformation of the cell membrane caused by the mechanical force for opening or closing the extracellular cap-like structure [31]. Piezo1 upregulation has been discovered in multiple human malignancies, like prostate, breast, and gastric cancer, facilitating tumor angiogenesis and growth [32,33]. Likewise, highly expressed Piezo1 in the tumor cells also indicates poor prognosis in HCC patients [31,32]. However, studies on HCC-related Piezo1 are commonly concentrated on the liver matrix components, like the HSCs, and studies on the hepatocytes have given few clues on the mechanism by which Piezo1 functions in tumor formation or recurrence.

Consistent with online dataset mining, we found a gradual elevation of Piezo1 in the paracancerous liver tissues from the samples in our center, with a significance in accordance with the extent of liver fibrosis/cirrhosis. As observed, the short-term tumor recurrence after surgery was significantly correlated with both the stratified Piezo1 upregulation and the attenuation of polyploid hepatocytes. We suggest that Piezo1 may modulate and impact the hepatocyte polyploid homeostasis.

Followingly, we examined the exact biological effect of the activated or upregulated Piezo1. In reference to a reliable procedure published for studying the cytoskeleton architecture [34], we succeeded in reproducing a stiffened environment to culture the hepatocytes in vitro. Interestingly, hard stiffness incubation not only changed the morphological phenotype of the hepatocytes as expected on the point of cytoskeletal plasticity, but also efficiently upregulated the expression of Piezo1. Meanwhile, we introduced the agonist Yoda1 to seek the potential effects of Piezo1 on hepatocyte polyploid homeostasis.

We note that either highly expressed Piezo1 or the activation of this cation channel induced a significant influx of Ca^2+^ into the cytoplasm. We suggest that the tremendous influx of Ca^2+^ mediates the sharp decline of the binuclear polyploid hepatocytes.

The actin-binding Anillin plays a pivotal scaffold protein role in cell growth and migration, engaged in the process of cell division and the failure of cytokinesis, by structurally linking to the actomyosin ring from the cell membrane to the contractile-located RhoA [35]. Verified in our previous study, Anillin is abnormally overexpressed in HCC tissues, correlated with unsatisfactory outcomes even after radical surgery [36]. In addition, the following investigation further discovered a gradual upregulation of Anillin in the paracancerous tissues from a lower level in the non-fibrosis tissues to a higher level in the stiffened tissues. Here, we suppose that the expression changes in either Piezo1 or Anillin under the background of liver matrix stiffness are consistent.

To confirm the speculation, we explored the hepatocytes treated by stiffening gels or Yoda1. These independent treatments gave out two possibilities, either to stimulate the hepatocytes for an expression increase in Piezo1 through the higher stiffness environment, or to activate Piezo1 into an open status of the ion channel. Both of these treatments led to a similar remarkable influx of Ca^2+^, which indicated that the activation of Piezo1 was induced functionally in the hepatocytes. Consequently, as the flow cytometry assay demonstrated, a rapid decline of the binuclear polyploid hepatocytes and a significant decrease in ABH were observed. We believe that the influx of Ca^2+^ mediated by the activation of Piezo1 exerts an important function in the impairment of polyploidy homeostasis. Among the Ca^2+^-related intracellular signaling, we noticed RUNX2 as a candidate to intensively investigate, even though the previous study on it in HCC is limited compared with the other solid malignancies. RUNX2 belongs to the RUNX transcription factor family, responsible for the process of bone and chondrocyte differentiation, and has been reported to be abnormally overexpressed in multiple human malignancies, such as pancreatic cancer and breast cancer, promoting cell proliferation, metastasis, and angiogenesis [37]. Thus, we interrogated the expression change in RUNX2. Exceeding our expectations, according to the literature and the results obtained by our team, there was no significance in RUNX2 expression concerning HCC [38]. However, in the following experiment, we observed an interesting nuclear translocation of RUNX2 in the hepatocytes when the cells were incubated on the stiffened gels or treated with Yoda1. We believe that the activation of Piezo1 participates in the nuclear translocation of RUNX2 via the influx of Ca^2+^.

Based on this, we investigated the relationship between RUNX2 and Anillin. Since all the clues in our study highlighted the upregulation of Anillin either in HCC tumorigenesis or the intrahepatic microenvironment stiffened, we predicted and screened the potential transcription factors upstream of Anillin. Intriguingly, RUNX2 was recommended as one of the predicted candidates binding to the promoter of the Anillin gene. The following ChIP assay was conducted to validate the direct interaction between RUNX2 and the Anillin gene promoter. To confirm the exact regulating effect of RUNX2 on Anillin, we further introduced high expression of RUNX2 in PHHs via lentiviral transduction. In accordance with our expectation, upregulating RUNX2 efficiently increased the transcription of ANLN mRNA, and the proportion of binuclear polyploid hepatocytes was decreased as credible evidence of the impairment of hepatocyte polyploid homeostasis.

Taken together, the overexpression and activation of Piezo1 induce a functional influx of Ca^2+^ into hepatocytes and lead to the essential nuclear translocation of RUNX2, which efficiently activates Anillin gene transcription and promotes attenuation of polyploid hepatocytes. Conditionally, the stiffened liver matrix is a crucial microenvironment component that stimulates Piezo1 expression at the precancerous stage of HCC. Severe intrahepatic fibrosis or cirrhosis generates the intrinsic mechanical signal, and initiates a mechanosensitive Piezo1/RUNX2/Anillin axis. As a result, this activated axis is associated with an efficient impairment of polyploid hepatocyte homeostasis and may contribute to the tumor formation of HCC. The findings of this study not only complement the probable mechanism concerning HCC tumorigenesis and tumor formation under the stiffened liver matrix, but also provide opportunities for intensively understanding the influence on hepatocyte polyploid attenuation in the pathological impaired microenvironment, which may help to create innovative strategies for HCC prevention and control. Also, several limitations should be acknowledged. This study was based on a single-center retrospective cohort with a relatively limited sample size, and further validation in larger multicenter prospective cohorts is needed. In addition, liver matrix stiffness was mainly evaluated according to pathological fibrosis/cirrhosis status rather than direct biomechanical measurement. Although the in vitro stiffness model allowed us to examine hepatocyte responses to mechanical cues, it cannot fully reproduce the complex hepatic microenvironment in vivo. Moreover, other mechanotransduction pathways, such as YAP/TAZ, integrin/FAK, RhoA/ROCK signaling, and cytoskeletal remodeling, may also be involved in stiffness-induced hepatocyte responses. Finally, the causal role of Piezo1 in vivo remains to be confirmed using hepatocyte-specific Piezo1 knockout models. Future studies are needed to further clarify how liver matrix stiffening contributes to polyploid attenuation and HCC formation.

## 4. Materials and Methods

### 4.1. Paracancerous Sample Preparation

A total of 142 paired samples, including tumor tissues and paracancerous liver tissues 1 cm close to the tumor margins, were collected as a cohort for exploring the relationship between liver matrix stiffness and the relevant parameters of HCC phenotypes. Another 43 cases of normal liver tissues without fibrosis or cirrhosis from patients with benign lesions like hemangiomas or hepatic cysts were collected simultaneously. All cases had received radical surgery treatment with no preoperative treatment and no adjuvant treatment at the Department of General Surgery, Ruijin Hospital, Shanghai Jiao Tong University School of Medicine (2018 to 2019). Informed consent was obtained and the study was approved by the Ethics Committee of Ruijin Hospital, Shanghai Jiaotong University School of Medicine, following the Declaration of Helsinki (2021-421). Hematoxylin-eosin (HE) staining was conducted for basic pathological evaluation. The clinicopathological parameters related to the tumor phenotypes, including preoperative alpha-fetoprotein (AFP), vitamin K Absence-II (PIVKA-II) value, tumor size, grades, and other relevant varibles, were collected. Tumor recurrence was defined according to the local recurrence postoperation within 2 years. Paracancerous liver matrix stiffness was graded according to the METAVIR scoring criteria for liver fibrosis/sclerosis (F1~F4). And the paracancerous samples were divided into the ‘lower stiffness’ group (F1~F2, n = 78) and the ‘higher stiffness’ group (F3~F4, n = 64) (two experienced pathologists were assigned independently).

### 4.2. Cell Culture

THLE-2 immortal hepatocytes were purchased from ATCC (CRL-2706) and cultured in the Endothelial cell medium (ScienCell, Carlsbad, CA, USA). Primary hepatocytes were isolated from 6- to 8-week-old male mice using the collagenase (Gibco, Grand Island, NE, USA) perfusion method. The isolated hepatocytes were resuspended in DMEM (Corning, Corning, NY, USA) supplemented with 10% FBS (Biological Industries, Kibbutz Beit Haemek, Israel) and penicillin–streptomycin solution (Gibco, Grand Island, NE, USA). The primary human hepatocytes (PHHs) were plated in 6-well plates at a density of 2.5 × 10^5^ cells/well and cultured for 48 h. Yoda1 (TOCRIS, Bristol, UK) was added to the medium at a final concentration of 15 nM for the indicated time period.

### 4.3. Datasets from the GEO Database Recruitment

The GSE45114, GSE14520, GSE14323, and GSE6764 datasets from the Gene Expression Omnibus (GEO: https://www.ncbi.nlm.nih.gov/geo/ (accessed on 10 November 2025)) database were collected. Platforms of GSE45114, GSE14520, GSE14323, and GSE6764 datasets were GPL5918, GPL3921, GPL571, and GPL570 respectively. The microarray data were preprocessed through the R language, version 4.4.1, and normalized by two specialized bioinformatics analysts.

### 4.4. Mechanical Environment Gel Preparation for Cell Culture

To prepare the mechanical environment gel for cell culture, we referred to the method of preparing the gel described by Gaudenz et al. [34]. Stiff substrates were generated by coating tissue culture dishes with collagen type I (Coring, Coring, NY, USA). When coating, a sufficient amount of diluted collagen solution (50 μg/mL in PBS) was added to fully cover the surface of a culture dish before incubating it for 2 h at 37 °C. The remaining solution was aspirated, and the dish was rinsed with PBS before seeding cells. Soft substrates were generated by polymerizing a collagen type I gel (Corning, Coring, NY, USA) on a 6-well plate. A collagen gel solution was made by chilling the collagen solution with 10× PBS and NaOH (0.1 N), adjusting the pH of the mixture to approximately 7.0. The mixture was added to the six-well plate and incubated overnight or until completion of the gelation at 37 °C. When collecting cells from the soft substrates, the cells were first washed with pre-warmed Hank’s Balanced Salt Solution (HBSS) (Thermo Fisher Scientific, Waltham, MA, USA), and the collagen gels were digested using pre-warmed collagenase type I (Gibco, USA) at 37 °C for 1 h.

### 4.5. RNA Extraction and Quantitative PCR

Total RNA was isolated according to the instructions of the TRIzol reagent (Invitrogen, Carlsbad, CA, USA). The first-strand cDNA was synthesized by using the High-Capacity cDNA Reverse Transcription Kit (ABI, Foster City, CA, USA), and the primers of Piezo1, RUNX2, and Anillin were prepared (Laite Biotech, Shanghai, China) (Supplementary Table S1). Real-time quantitative polymerase chain reaction (RT-qPCR) was conducted according to the TaqMan Gene Expression Assays protocol (ABI, USA).

### 4.6. Calcium Imaging and Quantification

The hepatocytes were pretreated with DMSO or the Piezo1 agonist Yoda1 before calcium imaging, and the treatments were maintained throughout Fluo-4 AM (Beyotime, Shanghai, China, S1061S) labeling and imaging. The cells were then stained in assay buffer for 20 min at 37 °C, followed by washing three times gently with PBS, and then the cells were mounted with a fluorescent mounting medium (YEASEN, Shanghai, China) and examined under a confocal microscope (Leica, Wetzlar, Germany).

### 4.7. HE Staining, Western Blot Assay, and Immunohistochemistry Assay

Following the conventional pathological exploration, H&E-stained slides were prepared. Two experienced pathologists were assigned to review the slides independently following the method in our previous study for histopathologic evaluation of ABH, combined with computer-assisted assessment we reported recently [11,12].

The Western blot analysis and the immunohistochemistry (IHC) assay were carried out according to the previous description [39]. The antibodies against Piezo1, RUNX2, and Anillin were purchased from Proteintech, China, and used according to the manufacturer’s instructions. The samples were separated according to the staining intensity grade for two groups: low staining (0~1+) and high staining (2+~3+).

### 4.8. Plasmid Preparation and Cell Transfection

The lentiviral vectors pLKO.1 containing shRNA was transfected into cultured hepatocytes at the exponential phase (Laite Biotech, Shanghai, China) to selectively suppressPiezo1, and the control vectors were assigned. The transfected cells were selected by using a medium mixed with 400 μg/mL G418 (Santa Cruz Biotechnology, Dallas, TX, USA). Simultaneously, the lentiviral vector pLV (Addgene, Watertown, MA, USA) was applied for ectopically expressing RUNX2 (pLV-RUNX2) for the rescue experiments, and the pLV-Null was set for control.

### 4.9. Isolation of Nuclear and Cytoplasmic Extract

The nuclear extraction was prepared by using the nuclear and cytoplasmic protein extraction kit (MeilunBio, Dalian, China) according to the product instructions. The cells were suspended in CER A, followed by the addition of CER B. The fraction of cytoplasmic extract supernatant was transferred to a pre-chilled tube, and the insoluble pellet fraction containing crude nuclei was resuspended in nuclear extraction reagent for vortexing, incubation on ice, followed by centrifugation. The resulting supernatant, constituting the nuclear extract, was used for the subsequent experiments.

### 4.10. Immunofluorescent Staining

The cells were fixed with 4% paraformaldehyde for 10 min, permeabilized with 0.1% Triton X-100 for 10 min and blocked with blocking buffer, followed by incubation with Piezo1, RUNX2, or ATP1A1 (Proteintech, Chicago, IL, USA) antibody overnight at 4 °C. The cells were rinsed in PBS three times and incubated with Alexa Fluor 555-conjugated (Life Technologies, Shanghai, China) or Alexa-Fluor-488-labeled goat anti-rabbit-IgG (Life Technologies, Carlsbad, CA, USA) for 1 h at room temperature in the dark. Incubation with DAPI (YEASEN, Shanghai, China) at room temperature for 10 min was used for nuclei staining. After being washed extensively with PBS, the cells were mounted with a fluorescent mounting medium (YEASEN, Shanghai, China) and examined under a confocal microscope (Leica, Wetzlar, Germany).

### 4.11. Flow Cytometry Assay

The flow cytometry analysis of cell ploidy was carried out following the collection of the cells fixed with 75% ethanol (containing 1 mM EDTA). The cells were washed with PBS (containing 1 mM of EDTA), and the fixative was removed from cells before proceeding with cell staining. Flow cytometry samples were prepared, each containing 1 × 10^6^ cells in suspension. The samples were centrifuged and the supernatant was discarded, leaving a pellet of cells in each sample tube. A total of 0.5 mL FxCycle™ PI/RNase Staining Solution (Invitrogen, Carlsbad, CA, USA) was added to each flow cytometry sample. All the samples were incubated for 15 min at room temperature, protected from light. Sample analysis was conducted without washing, using the 488 nm or 532 nm excitation, and emission was collected by using a 585/42 bandpass filter or equivalent. For flow cytometric analysis of hepatocyte ploidy, cell debris was first excluded based on FSC/SSC parameters. Cell aggregates and doublets were then excluded using FSC/SSC-B gating, and only single-cell populations were retained for DNA content analysis. Subsequently, cell populations were distinguished according to PI fluorescence intensity and used to evaluate hepatocyte ploidy status.

### 4.12. Statistical Analysis

Statistical analysis was carried out by using SPSS 20.0 and GraphPad Prism 10. *p* values were calculated using an unpaired Student’s *t*-test and Fisher’s exact test. Differences were considered statistically significant at *p*-values < 0.05.

## Figures and Tables

**Table 1:** Correlations of fibrosis, E2F7, and Anillin with clinicopathological characteristics in 142 HCC patients. The correlation between fibrosis, E2F7, Anillin, and clinicopathological characteristics were analyzed using Fisher’s exact test or χ^2^ test, as appropriate. The clinicopathological characteristics include age, sex, tumor, TNM stage, recurrence, tumor encapsulation, tumor microsatellite, venous invasion, HBsAg, AFP, PIVKA-II and ABH. The data for AFP and PIVKA-II were collected immediately prior to surgery. (* Statistical significance: * *p* value < 0.05, ** *p* value < 0.01, *** *p* value < 0.001.).

	Fibrosis		*p*-Value	E2F7 Level		*p*-Value	Anillin Level		*p*-Value
	0–2	3–4		Low	High		Low	High	
	n = 68	n = 74		n = 32	n = 110		n = 40	n = 102	
**Age (years)**									
≤50	43	51	0.475	19	75	0.398	26	68	0.846
>50	25	23		13	35		14	34	
**Sex**									
Male	47	48	0.591	22	73	0.99	26	69	0.843
Female	21	26		10	37		14	33	
**Dimeter (cm)**									
≤5	40	42	0.803	26	56	0.002 **	32	50	<0.001 ***
>5	28	32		6	54		8	52	
**TNM stage**									
I-II	41	35	0.121	26	50	<0.001 ***	29	47	0.005 **
III-IV	27	39		6	60		11	55	
**Recurrence (m)**									
>24	58	49	0.008 **	29	78	0.023 *	35	72	0.035 *
≤24	10	25		3	32		5	30	
**Tumor encapsulation**									
Absent	37	34	0.314	19	52	0.315	17	54	0.351
Present	31	40		13	58		23	48	
**Tumor microsatellite**									
Absent	32	32	0.648	20	44	0.028 *	28	36	<0.001 ***
Present	36	42		12	66		12	66	
**Venous invasion**									
No	36	38	0.850	22	52	0.044 *	28	46	0.009 **
Yes	32	36		10	58		12	56	
**HBsAg**									
Negative	17	6	0.006 **	6	17	0.785	13	10	0.002 **
Positive	51	68		26	93		27	92	
**AFP (ng/mL)**									
≤400	29	24	0.208	18	35	0.021 *	24	29	<0.001 ***
>400	39	50		14	75		16	73	
**PIVKA-II**									
Positive	44	58	0.070	15	87	<0.001 ***	23	79	0.023 *
Negative	24	16		17	23		17	23	
**ABH (%)**									
≤1.5	22	34	0.022 *	9	47	0.137	9	47	0.010 *
>1.5	46	40		23	63		31	55	

## Data Availability

Data from this study is not made public due to privacy regulations but is available on request from the authors via sending a message to Junqing Wang (E-mail: wangjunqingmd@hotmail.com).

## Supplementary Materials

The following supporting information can be downloaded at: https://www.mdpi.com/article/10.3390/ijms27114685/s1.

## Abbreviations

Hepatocellular carcinoma (HCC); Piezo-type mechanosensitive ion channel component 1 (Piezo1); RUNX Family Transcription Factor 2 (RUNX2); Anillin Actin Binding Protein (Anillin); the chromatin immunoprecipitation assay (ChIP assay).
