# Supplementary material for: Liver Matrix Stiffening Modulates Tumor-Associated Hepatocyte Polyploid Homeostasis via Piezo1/RUNX2/Anillin Mechanosensitive Axis
Source: Int J Mol Sci. 2026 May 22;27(11):4685. doi: 10.3390/ijms27114685 (PMC13256888; doi:10.3390/ijms27114685)
Supplement: Supplementary file 1 [file ijms-27-04685-s001.zip › Supplementary Materials.pdf]

Supplementary Materials

Table S1.

The primers for RT-qPCR assay or ChIP assay

Genes	Forward	Reverse
Piezo1	5’- TCCTCAACCACATGGTCACG-3’	5’- GCGATCTCGGTGAAGACGAT -3’
KLF13	5’- CGCCTCACAAACAACCACAG -3’	5’- TCACTGTGCTGAAGAGGCTG -3’
Anillin	5’- TCCTCTGCAAGTGGAGCATC -3’	5’- TCACGTATCACTTTAGGTTCTGT -3’
Primer 1	5’-TGAACTACCGGGGGTTAAGA-3’	5’-TACACACACAGACACGCCC-3’
Primer 2	5’-GGGGGTTAAGAATGAAAGGA-3’	5’-GCCCCAAATACATACACACC-3’

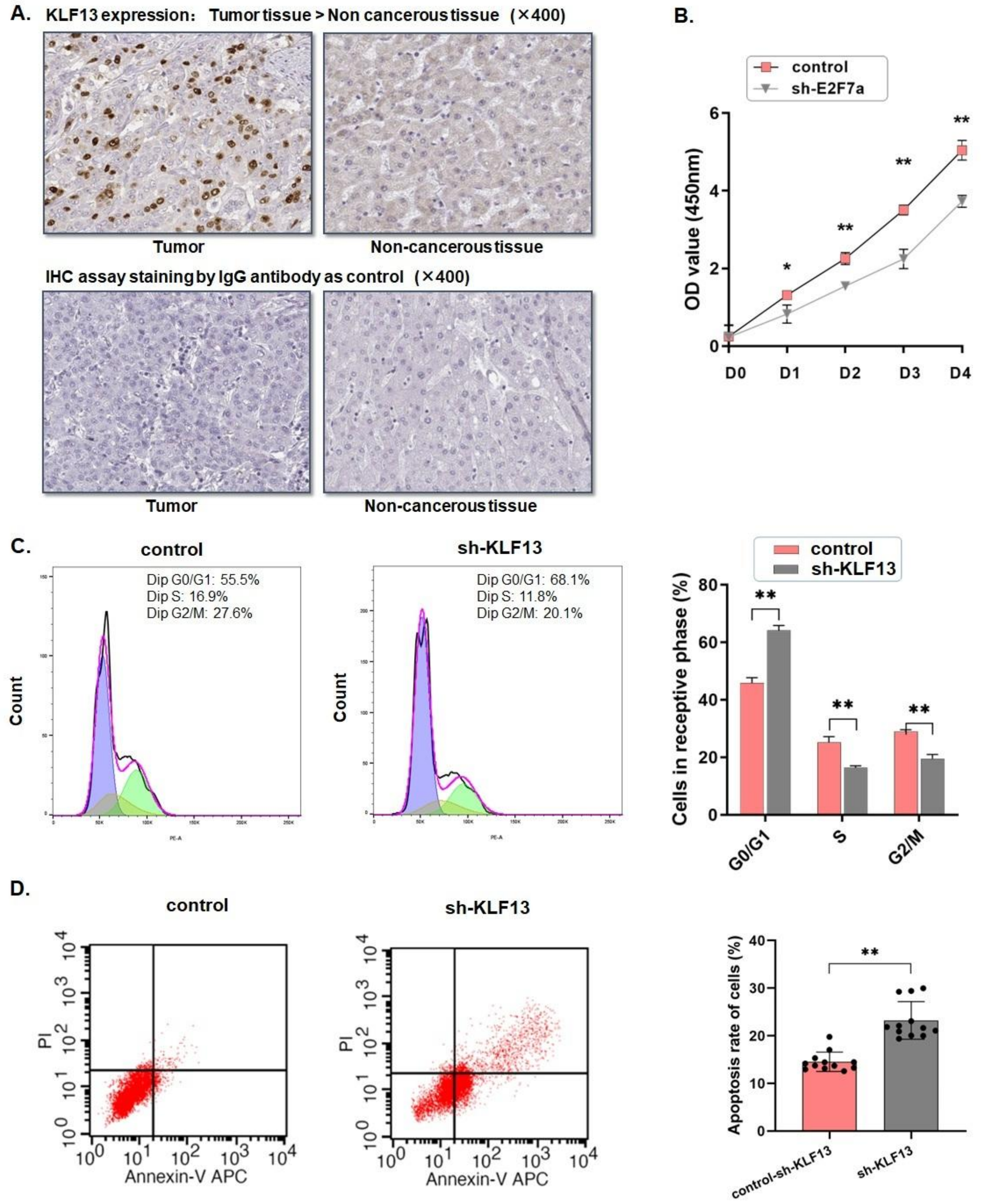

Figure S1. KLF13 expression profile in HCC and the in vitro experiment after depleting KLF13 in the cell line

A. The representative graphs of immunohistochemistry analysis (400×) of HCC patients' specimens. Specimens were regarded as the control by staining IgG antibodies. KLF13 expression in tumor tissues was significantly higher than in adjacent non-cancerous tissues. **B.** The CCK8 assay was applied. The HCC cell proliferation (HepG2) was significantly suppressed by depleting KLF13 (*$P < 0.05$, **$P < 0.01$). **C.** Flow cytometry was conducted for detecting the cell cycle. The

representative dot plots show that the cell cycle of HCC cells was arrested in the G0/G1 phase by depleting KLF13. The results are means of three independent experiments ±SD. (**P< 0.01). **D.** Cell apoptosis rate was detected by flow cytometry exploration. The representative histograms describe that the cell apoptosis rate of HCC cells was significantly increased in HepG2 cells after KLF13 depletion. The results are means of three independent experiments ±SD. (**P< 0.01).

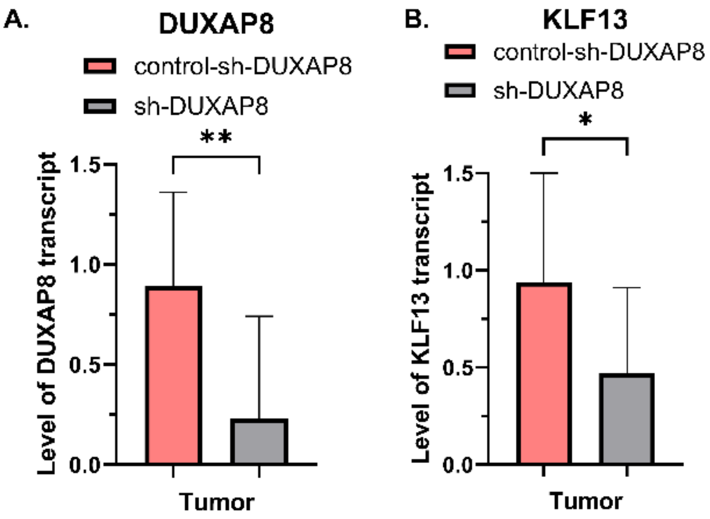

Figure S2. DUXAP8 and KLF13 expression level in xenograft tumor

A. RT-qPCR assay was performed to validate the knockdown of DUXAP8 in the xenograft tumor. (**P<0.01). **B.** RT-qPCR assay demonstrates the decline of KLF13 after DUXAP8 depletion *in vivo* (*P<0.05).
